# Impact of quantitative pulmonary emphysema score on the rate of pneumothorax and chest tube insertion in CT-guided lung biopsies

**DOI:** 10.1038/s41598-020-67348-0

**Published:** 2020-07-03

**Authors:** Dorothea Theilig, David Petschelt, Anna Mayerhofer, Bernd Hamm, Bernhard Gebauer, Dominik Geisel

**Affiliations:** Department of Diagnostic and Interventional Radiology, Charité – Universitätsmedizin Berlin, corporate member of Freie Universität Berlin, Humboldt-Universität zu Berlin, and Berlin Institute of Health, Augustenburger Platz 1, 13353 Berlin, Germany

**Keywords:** Outcomes research, Clinical trial design

## Abstract

The aim of this study was to evaluate the risk of pneumothorax and need for chest tube insertion in CT-guided lung biopsies and identify predictors focusing on pulmonary emphysema determined with quantitative computed tomography. To that end, we retrospectively analysed the incidence of pneumothorax and chest tube insertion in 371 CT-guided lung biopsies with respect to the quantitative emphysema score determined with the density mask technique. Other possible impact factors considered were lesion diameter, length of biopsy pathway within the lung parenchyma, lung lobe, needle size, puncture technique, patient positioning and interventionalist’s level of experience. Quantitative emphysema scores of the lung were significantly higher in patients who developed instant pneumothorax (27%, *p* < 0.0001), overall pneumothorax (38%, *p* = 0.001) and had chest tube insertion (9%, *p* = 0.006) compared to those who did not when analysed with the Mann–Whitney U-test. In logistic regression analysis with inclusion of the other possible impact factors, the quantitative emphysema score remained a statistically significant predictor for all three output parameters. This was confirmed with least absolute shrinkage and selection operator (Lasso) regression analysis. In conclusion, quantitatively determined pulmonary emphysema is a positive predictor of the pneumothorax rate in CT-guided lung biopsy and likelihood of chest tube insertion.

## Introduction

Incidentally detected pulmonary nodules often pose a diagnostic dilemma. In its 2017 guidelines, the Fleischner society recommends either positron emission tomography/computed tomography (PET/CT) or biopsy for an incidentally found singular, solid pulmonary nodule greater than 8 mm in diameter to rule out malignancy^1^. Furthermore, in patients with more than one primary malignancy, the origin of pulmonary metastasis is not apparent from diagnostic imaging. In such a scenario, treatment planning also requires tissue sampling for pathologic clarification.

CT-guided biopsy of pulmonary nodules has become a standard procedure for tissue sampling. Compared to navigational bronchoscopy with biopsy, percutaneous CT-guided biopsies are less expensive and have a higher diagnostic yield in the assessment of peripheral pulmonary nodules^2,3^. Compared to surgery, percutaneous CT-guided lung biopsies are less invasive and reduce the amount of anaesthesia required. Nevertheless, CT-guided lung biopsies also come along with risks^4^. In addition to the risks shared by all CT-guided interventions like infection, bleeding and nerve injury, lung biopsies have specific risks including pneumothorax and inadvertent creation of a fistula between a bronchus and blood vessel with possibly fatal gas embolism^5^. Pneumothorax is by far the most frequent complication of lung biopsies.

In the literature, the frequency of pneumothorax associated with lung biopsy is reported to range from 9 to 54%^6^, among them a large retrospective study of 9,783 lung biopsies with a rate of 35%^4^. The reported rate of patients requiring chest tube placement ranges from 2 to 15%^6^.

There are a number of studies investigating factors which might have an impact on the occurrence of pneumothorax and the need for chest tube insertion following lung biopsy with pulmonary emphysema being one of them^6–12^. In these studies, pulmonary emphysema was assessed visually, which is rather subjective and time-consuming as the lung is a large organ covering many slices in a CT scan.

Over the last decade, quantitative CT has emerged as an objective method to quantify emphysematous parenchymal changes by identifying pulmonary areas of low attenuation in thin-section chest CT scans with a suitable software algorithm. This so-called density mask technique is described in detail elsewhere^13^. To date, there are only a few studies, each including only a moderate number of study patients, that have employed this quantitative method to analyse the associated risk of pneumothorax and while there is more evidence in favour of quantitative emphysema being a risk factor^14,15^ there is also some against it^16^.

The aim of this study was to elucidate the rate of pneumothorax in our department and to identify risk factors focusing on pulmonary emphysema as determined with quantitative CT. For that purpose, we assessed pulmonary emphysema on thin-section CT scans acquired within a year prior to the biopsy using a dedicated software package. In order to include all relevant risk factors besides pulmonary emphysema the pertinent literature was thoroughly reviewed prior to data acquisition. The identified risk factors which were included into the analysis comprised needle size, puncture technique, lesion size, length of puncture tract in lung parenchyma, lung lobe harbouring the lesion, positioning of the patient during the procedure and level of experience of the interventionalist^7,8,17,18^.

## Materials and methods

The study was approved by the institutional review board (Charité's Ethics Committee—Charitéplatz 1, 10117 Berlin, Germany) and all methods were carried out in accordance with relevant guidelines and regulations. The ethic committee waived informed consent requirements for this retrospective study.

### Study design

**Figure 1:**
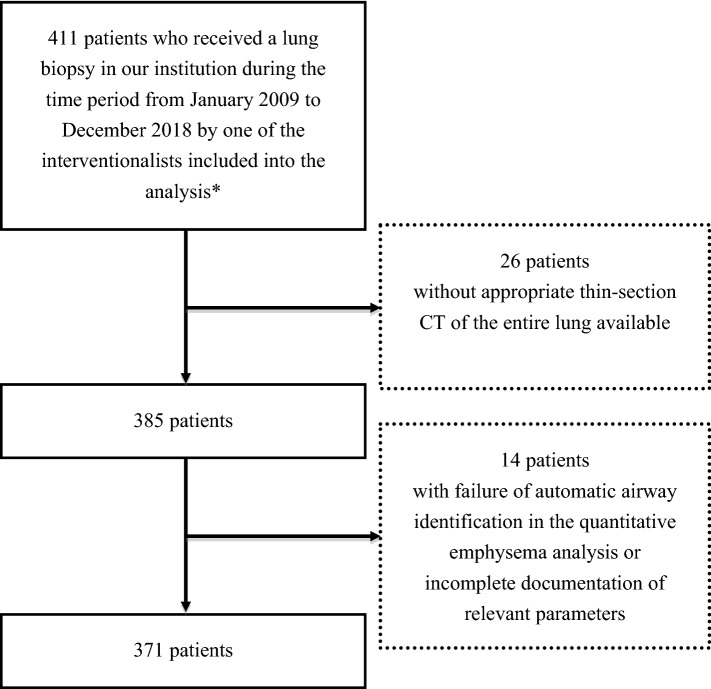
Flow chart of patients included into the study. * Inclusion criteria for interventionalists were being a board-certified physician training to become a radiologist and having started the interventional career with CT-guided interventions during the stated time period. A random selection of interventionalists meeting these criteria was performed.

We retrospectively analysed 371 CT-guided lung biopsies performed at our institution on the same CT scanner over a 10-year period from 2009 to 2018. A flow chart of patients included into the study is provided in Fig. 1. A thin-section chest CT scan acquired within a year prior to the biopsy was used to determine a quantitative pulmonary emphysema score with the density mask technique. Information on lung lesion diameter, length of biopsy pathway within the lung parenchyma measured from the pleural puncture site to the edge of the pulmonary lesion along the needle path, lobe of lesion location and positioning of the patient during the procedure was retrieved from the dose reports and fluoroscopy images, both stored in the picture archiving and communication system (PACS). The needle size and puncture technique were retrieved from the written reports, and the number of lung biopsies previously performed by each interventionalist was determined from the radiological information system (RIS) and used as a surrogate for his or her level of experience.

Fluoroscopy images of the lung biopsy procedure and the x-ray of the lung routinely performed 4 h after biopsy as well as any additional lung images acquired during the following week were screened for pneumothorax. If a pneumothorax was identified, follow-up images were further analysed to find out whether a chest tube was inserted to treat the pneumothorax or whether spontaneous resorption of the pneumothorax occurred. Findings were grouped into three categories: (1) “instant pneumothorax” for pneumothorax identified during the procedure, (2) “overall pneumothorax” for pneumothorax identified during the procedure or on follow-up images acquired within one week and (3) “chest tube insertion” for biopsy-related pneumothorax treated by chest tube insertion.

### CT fluoroscopy and interventional workflow

All CT-guided lung biopsies were performed on the same CT scanner, a Siemens Definition AS with a 32-row detector and a z-flying focal spot. The so called quick-check technique with intermittent 5-mm single-slice images taken was used. The quick-check technique is essentially analogous to conventional CT except for faster reconstruction times and manual table positioning by the radiologist^19^. The image acquisition parameters were set at 30 mAS and 120 kV unless the interventionalist deemed it necessary to alter these parameters. All biopsies investigated in this study were performed by radiologists who started their interventional career during the study period. This setting allowed us to count the lung biopsies performed by the interventionalist before the one in question and that number was used as a surrogate for his or her level of experience at the time of the study biopsy.

A conventional CT scan of the region of interest was obtained prior to the intervention and was used to plan the best way to access the lesion. Basic principles observed for pathway planning as best as possible were choosing an angle perpendicular to the pleura, not crossing a lung fissure, avoiding large bronchi or blood vessels, minimising the length of the puncture pathway and passing at the upper edge of ribs in order to minimise the likelihood of costal nerve or vessel injury as recommended in the literature^20,21^.

Routine preparation before the biopsy procedure included lead shielding and sterile covering, followed by administration of local anaesthesia.

In the majority of cases, a coaxial technique was employed using a coaxial needle (Co-axial Introducer Needle, Argon Medical Devices, Athens, TX 75751, USA) combined with a cutting needle (Quick-Core Biopsy Needle, Cook Medical, Bloomington, IN 47404, USA) and 2 samples, each 2 cm in length, were taken. In most cases, a 19 Gauge coaxial needle was combined with a 20 Gauge cutting needle. Less frequently, an 18- or 20-Gauge cutting needle was used alone, taking only one 2-cm sample. For our analysis, we retrieved the needle size and technique from the written reports.

### Emphysema score

Replacement of normal lung parenchyma with an attenuation of approx. -850 HU on inspiratory CT scans by air-filled spaces with lower attenuation is characteristic for emphysematous lung destruction. The density masque technique has emerged as an objective and quantitative method to determine the degree of pulmonary emphysema. Most commonly, a threshold of − 950 HU is used to separate emphysematous from normal lung parenchyma^22,23^. After automatic identification of the lung and airway segmentation voxels within the lung below − 950 HU are automatically identified for calculating the emphysema score. The emphysema score is defined as the number of voxels below − 950 HU divided by the total number of voxels of the lung. The quantitative emphysema score is therefore referred to as %LAA-950 (low-attenuation areas less than − 950 Hounsfield Units) from here onwards. Parameters of the CT scans used in this study for quantitative emphysema analysis were as follows: 120 kV, automatic dose modulation (range 100–500 mA), standard kernel, ≤ 1.25 mm slice thickness and acquisition in inspiration. Several software packages are now available for this analysis; in this study, we used the software package from General Electrics (AW Server 3.2, Ext. 1.2, 2016).

### Statistics

The Shapiro–Wilk test was used to test for normal distribution. The Mann–Whitney U-test was used as a nonparametric test to compare continuous variables between two groups. The Chi-square test was employed to compare categorical variables.

Binomial logistic regression analysis was used to identify factors with an impact on the likelihood of lung biopsy-related instant pneumothorax, overall pneumothorax and chest tube insertion. Additionally, least absolute shrinkage and selection operator (Lasso) regression with control variables to be selected by lassos and tenfold cross-validation was performed. Measures of performance to predict instant pneumothorax, overall pneumothorax and pneumothorax with chest tube insertion was performed with fivefold cross validation testing the following models: Fine Tree, Medium Tree, Coarse Tree, Logistic Regression, Gaussian Naive Bayes, Kernel Naive Bayes, Linear SVM (Support Vector Machines), Quadratic SVM, Cubic SVM, Fine Gaussian SVM, Median Gaussian SVM, Coarse Gaussian SVM, Boosted Trees, Ensembled Trees, RUSBossted Trees.

A *p* value of < 0.05 was considered statistically significant. Statistical analysis was performed with Stata/MP Version 16 (StataCorp, College Station, TX, USA) and Matlab 2020 (The MathWorks, Natick, MA, USA).

## Results

Among the 371 lung biopsies retrospectively analysed in this study, we identified 102 patients (27%) with an instant pneumothorax and 140 patients (38%) with overall pneumothorax. Thirty-five patients (9%) had a pneumothorax with a size and symptoms requiring chest tube insertion while 231 patients (62%) did not develop a pneumothorax at all. Table 1 summarises descriptive statistical results in the total study population and these four subgroups and effects of possible impact factors on the occurrence of lung biopsy-related pneumothorax.

**Table 1:** Descriptive statistics.

	Total	Instant pneumothorax	Overall pneumothorax	Chest tube required	No pneumothorax	*p* value*^1^ Overall versus no pneumothorax	*p* value*^1^ Chest tube versus no chest tube
Number	371	102 (27%)	140 (38%)	35 (9%)	231 (62%)	–	–
Emphysema score (%LAA-950)	3.06 ± 6.31	5.06 ± 8.14	4.22 ± 7.26	5.81 ± 8.26	2.36 ± 5.56	0.001 *	0.006*
Lung parenchyma distance (mm)	20 ± 20	23 ± 22	26 ± 21	34 ± 29	16 ± 18	< 0.001*	< 0.001*
Lesion diameter (mm)	32 ± 26	26 ± 16	26 ± 15	26 ± 13	36 ± 30	< 0.001*	0.150
**Lung lobe**							
UL	191 (52%)	44 (43%)	75 (54%)	23 (66%)	116 (51%)	0.814	0.094
LL	158 (43%)	50 (49%)	57 (41%)	9 (26%)	101 (44%)		
ML	20 (5%)	8 (8%)	8 (6%)	3 (9%)	12 (5%)		
**Biopsy needle size**							
16 G	6 (2%)	0 (0%)	0 (0%)	0 (0%)	6 (3%)	0.156	0.661
18 G	118 (32%)	30 (30%)	46 (33%)	12 (36%)	72 (31%)		
20 G	244 (66%)	70 (70%)	92 (67%)	21 (64%)	152 (66%)		
**Puncture technique**							
Direct	107 (29%)	39 (38%)	46 (33%)	11 (31%)	61 (26%)	0.184	0.731
Co-axial	264 (71%)	63 (62%)	94 (67%)	24 (69%)	170 (74%)		
**Patient positioning**							
Prone	182 (49%)	57 (56%)	71 (51%)	12 (34%)	111 (48%)	0.452	0.120
Supine	171 (46%)	41 (40%)	65 (46%)	22 (63%)	106 (46%)		
Lateral	17 (5%)	4 (4%)	4 (3%)	1 (3%)	13 (6%)		
No of performed lung biopsies by interventionalist	17 ± 13	15 ± 11	16 ± 12	17 ± 12	17 ± 13	0.222	0.340

For categorical variables the percentage (%) and for continuous variables the standard deviation (± SD) is given. %LAA-950 percentage of low-attenuation areas of less than − 950 Hounsfield Units; *UL* upper lobe, *LL* lower lobe, *ML* middle lobe, *G* Gauge. *^1^*p* value of Chi-Square-Test for categorical variables and Mann–Whitney U-test for continuous variables. *p < 0.05.

**Figure 2:**
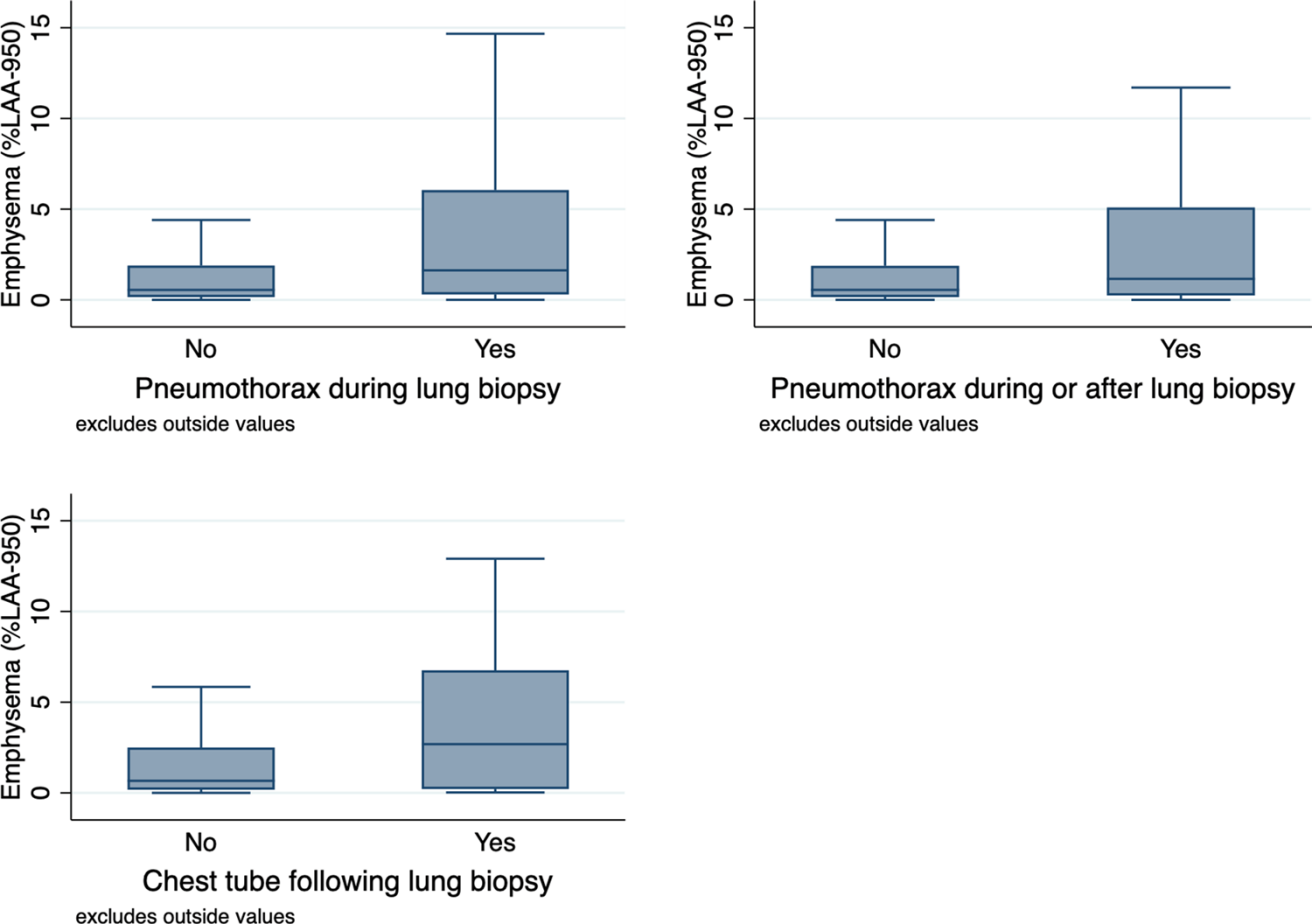
Boxplot graphs of quantitatively determined emphysema scores (%LAA-950) of patients with lung biopsy-related instant pneumothorax, overall pneumothorax and chest tube insertion versus patients without pneumothorax.

**Figure 3:**
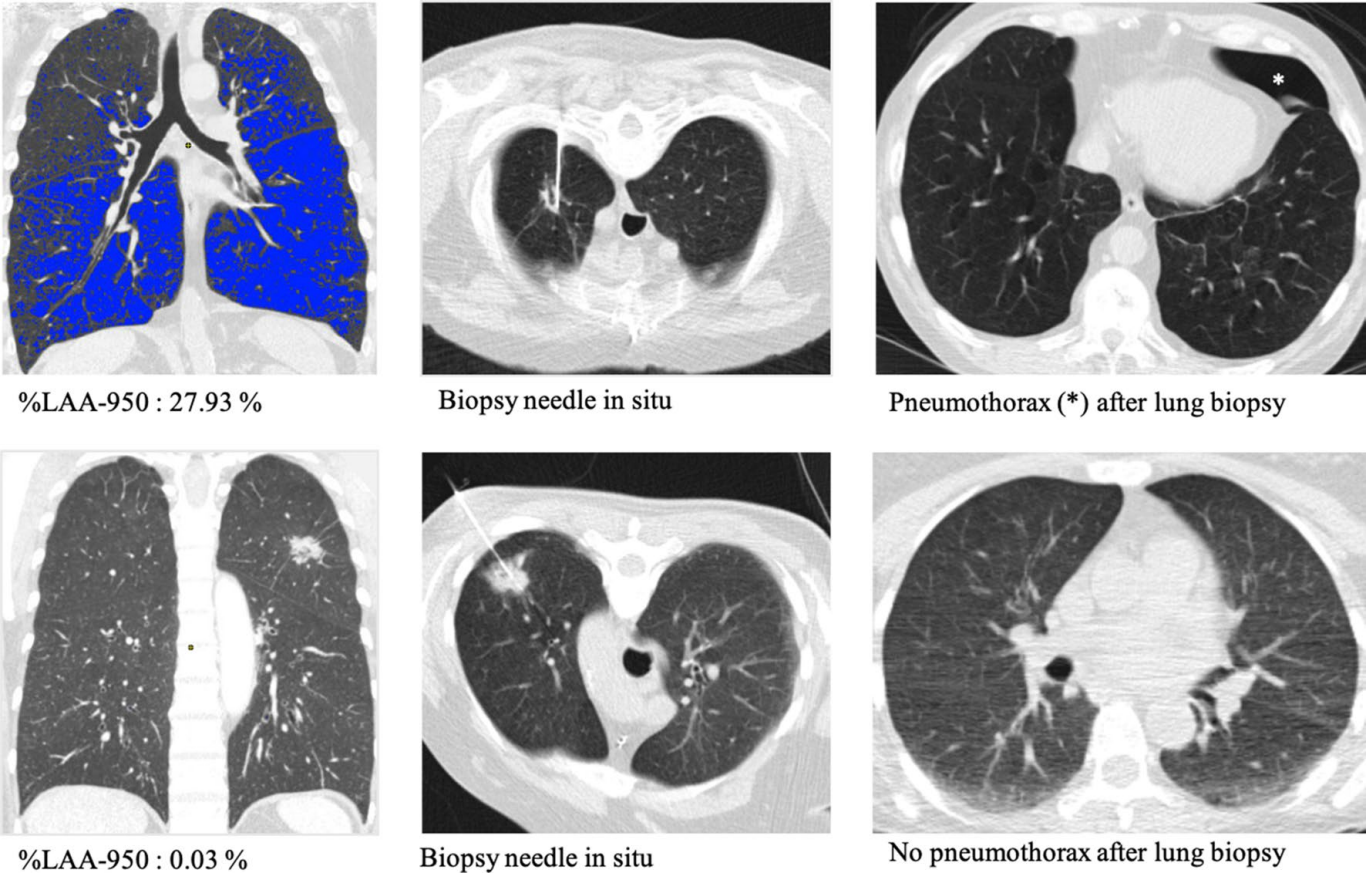
Images illustrating findings in two patients with CT-guided lung biopsy: patient A in row 1 with a high emphysema score and patient B in row 2 with a low emphysema score. Column 1 shows coronal plane CT images with automatic identification of voxels < − 950 HU depicted in blue and provides the resulting quantitative emphysema score (%LAA-950) generated with AW Server 3.2, Ext. 1.2, 2016 (https://www.gehealthcare.de/products/advanced-visualization/platforms/aw-server), column 2 shows axial CT-fluoroscopy images with the biopsy needle in situ, and column 3 shows axial CT-fluoroscopy images of the same site after biopsy with and without pneumothorax.

Quantitatively determined pulmonary emphysema scores (%LAA-950) were significantly higher in patients who developed an instant pneumothorax (*p* < 0.0001), a pneumothorax overall (*p* = 0.001) and had a chest tube inserted (*p* = 0.006) compared to those without pneumothorax in the Mann–Whitney U-test. Figure 2 shows boxplots of %LAA-950 in these three groups of patients. Corresponding imaging examples of two patients with CT-guided lung biopsy are provided in Fig. 3.

To account for other factors with a possible impact on the pneumothorax rate following lung biopsy binominal logistic regression was used. Other factors analysed were length of lung parenchyma punctured, lesion diameter, lung lobe harbouring the lesion, biopsy needle size, puncture technique (coaxial or direct), patient positioning (prone, supine, lateral) and the interventionalist’s level of experience in terms of the number of lung biopsies performed before. In binominal logistic regression analysis of these parameters, only the pulmonary emphysema score and the lung parenchyma length punctured had a statistically significant positive impact on the likelihood of developing a pneumothorax following lung biopsy in all three subgroups, namely instant pneumothorax, overall pneumothorax and chest tube insertion. In these three subgroups, the pulmonary emphysema score yielded *p* < 0.001, *p* = 0.003 and *p* = 0.002 while the lung parenchyma length punctured yielded *p* = 0.035, *p* < 0.001 and *p* < 0.001, respectively.

The results of binomial logistic regression analysis for instant pneumothorax are shown in Table 2a (*p* value < 0.0001), those for overall pneumothorax in Table 2b (*p* value < 0.0001) and those for chest tube insertion in Table 2c (*p* value = 0.0001).

**Table 2:** (a) Binomial logistic regression analysis of factors predicting lung biopsy-related instant pneumothorax. N = 358. *p* value of the model < 0.0001. (b) Binomial logistic regression analysis of factors predicting lung biopsy-related overall pneumothorax. N = 358. *p* value of the model < 0.0001. (c) Binomial logistic regression analysis of factors predicting pneumothorax with chest tube insertion after lung biopsy. N = 357. *p* value of the model = 0.0001.

	Odds ratio	95% confidence interval		Z-value	*p* value	Sig
**(a)**						
Emphysema score (%LAA-950)	1.075	1.034	1.117	3.660	< 0.001	**
Length of lung parenchyma punctured (mm)	1.014	1.001	1.027	2.100	0.035	*
Lesion diameter (mm)	0.977	0.961	0.994	− 2.710	0.007	**
Lung lobe						
UL	0.258	0.090	0.738	− 2.520	0.012	*
LL	0.398	0.134	1.181	− 1.660	0.097	
ML	1	–	–	–	–	
Biopsy needle size						
16 G	1					
18 G	1.076	0.616	1.878	0.260	0.798	
20 G	1	–	–	–	–	
Puncture technique						
Direct	1.808	1.057	3.092	2.160	0.031	*
Coaxial	1	–	–	–	–	
Patient positioning						
Prone	1.896	0.518	6.933	0.970	0.334	
Supine	1.309	0.348	4.929	0.400	0.690	
Lateral	1	–	–	–	–	
Interventionalist’s level of experience (number of performed lung biopsies)	0.978	0.958	1.000	− 1.980	0.047	

	Odds ratio	95% confidence interval		Z-value	*p* value	Sig
**(b)**						
Emphysema score (%LAA-950)	1.057	1.018	1.097	2.920	0.003	**
Length of lung parenchyma punctured (mm)	1.026	1.013	1.040	4.020	< 0.001	**
Lesion diameter (mm)	0.977	0.963	0.992	− 3.010	0.003	**
Lung lobe						
UL	0.583	0.211	1.610	− 1.040	0.298	
LL	0.527	0.182	1.525	− 1.180	0.237	
ML	1	–	–	–	–	
Biopsy needle size						
16 G	1					
18 G	1.491	0.894	2.488	1.530	0.126	
20 G	1	–	–	–	–	
Puncture technique						
Direct	1.378	0.826	2.298	1.230	0.219	
Coaxial	1	–	–	–	–	
Patient positioning						
Prone	2.473	0.693	8.831	1.390	0.163	
Supine	1.992	0.546	7.262	1.040	0.297	
Lateral	1	–	–	–	–	
Interventionalist’s level of experience (number of performed lung biopsies)	0.983	0.965	1.002	–1.790	0.074	

	Odds ratio	95% confidence interval		Z-value	*p* value	Sig
**(c)**						
Emphysema score (%LAA-950)	1.074	1.027	1.124	3.110	0.002	**
Length of lung parenchyma punctured (mm)	1.038	1.020	1.056	4.150	< 0.001	**
Lesion diameter (mm)	0.987	0.960	1.015	− 0.930	0.354	
Lung lobe						
UL	0.419	0.104	1.689	− 1.220	0.221	
LL	0.202	0.041	0.993	− 1.970	0.049	*
ML	1	–	–	–	–	
Biopsy needle size						
16 G	1					
18 G	1.340	0.590	3.044	0.700	0.485	
20 G	1	–	–	–	–	
Puncture technique						
Direct	1.326	0.558	3.153	0.640	0.523	
Coaxial	1	–	–	–	–	
Patient positioning						
Prone	0.811	0.086	7.661	− 0.180	0.855	
Supine	1.340	0.142	12.668	0.260	0.798	
Lateral	1	–	–	–	–	
Interventionalist’s level of experience (number of performed lung biopsies)	0.999	0.969	1.031	− 0.040	0.969	

%LAA-950 percentage of low-attenuation areas of less than − 950 Hounsfield Units; *UL* upper lobe, *LL* lower lobe, *ML* middle lobe, *G* Gauge—omitted because of collinearity; **p < 0.01; *p < 0.05.

In the logistic regression analysis of instant pneumothorax (Table 2a) and overall pneumothorax (Table 2b), lesion diameter showed a significant negative impact on the likelihood of developing instant pneumothorax (*p* = 0.007) and overall pneumothorax (*p* = 0.003), i.e., the larger the lesion the less likely a pneumothorax ensued. In addition, upper lobe localisation of the lesion had a significant negative impact on instant pneumothorax (*p* = 0.012), i.e., was less likely to result in a pneumothorax, and the direct puncture technique had a statistically significant positive impact on instant pneumothorax (*p* = 0.031), i.e., the direct puncture technique was more likely to result in instant pneumothorax.

Conversely, in the logistic regression analysis of chest tube insertion (Table 2c), lower lobe localisation of the lesion had a negative impact (*p* = 0.049).

The statistical significance of emphysema scores for the outcome parameters was confirmed with a Lasso regression model with control variables to be selected by lassos and tenfold cross-validation. This model yielded *p* = 0.001 for instant pneumothorax, *p* = 0.002 for overall pneumothorax and *p* = 0.001 for chest tube insertion (Table I of supplementary material). Additionally, measures of performance to predict instant pneumothorax, overall pneumothorax and pneumothorax with chest tube insertion was performed with fivefold cross validation and the resulting best models and their accuracy are given in Table 3.

**Table 3:** SVM: support vector machines; AUC: area under the curve.

	Instant pneumothorax	Overall pneumothorax	Pneumothorax with chest tube insertion
Model	Linear SVM	Coarse Gaussian SVM	Gaussian Naive Bayes
AUC	0.70	0.69	0.73
Accuracy	72.8%	62.3%	86.2%

Separate analysis of patients with %LAA-950 > 5% revealed an instant pneumothorax rate of 49% in this subgroup as opposed to 23% in patients with %LAA-950 ≤ 5% (Table II of supplementary material).

Separate analysis of the puncture technique with the Chi-square test confirmed the results of the logistic regression analysis with direct biopsy resulting in significantly more instant pneumothoraces (*p* = 0.014) than the coaxial puncture technique while no statistically significant difference was found between the two techniques with respect to overall pneumothorax (*p* = 0.184) and chest tube insertion (*p* = 0.731).

## Discussion

In this retrospective analysis of 371 lung biopsies we found rates of pneumothorax (38%) and chest tube insertion (9%) similar to those reported in the literature^4,6^.

On the other hand, our results clearly show that the quantitative pulmonary emphysema score (%LAA-950) has a significant impact on the likelihood of developing a pneumothorax in general and a pneumothorax necessitating chest tube treatment with higher emphysema scores increasing the risk of being afflicted by a pneumothorax and requiring chest tube treatment. To our knowledge, only a few studies have so far investigated quantitatively determined pulmonary emphysema as a predictor of pneumothorax following CT-guided lung biopsy. These studies included moderate numbers of patients and only one of them additionally evaluated chest tube treatment^14–16^. Two of these studies found the quantitative emphysema score to be a predictor of pneumothorax^14,15^ (n = 163 and n = 100) while one did not^16^ (n = 97). Our analysis of n = 371 validates quantitatively determined pulmonary emphysema as a predictor of pneumothorax and is the first study to show that it is also a positive predictor of chest tube insertion for pneumothorax, which is contrary to the findings of the much smaller study of Lendeckel et al.^15^. The rather large study population we analysed and the quantitative method we used to determine pulmonary emphysema make our findings more robust than published data obtained with qualitative methods as outlined in the Introduction section and which are inconsistent with respect to the effect of pulmonary emphysema on the occurrence of lung biopsy-related pneumothorax^21^.

In our study %LAA-950 was the strongest predictor of instant pneumothorax and the second strongest predictor of overall pneumothorax and chest tube insertion. The only other factor that also had an impact on all three output parameters analysed in this study, that is instant pneumothorax, overall pneumothorax and chest tube insertion, was the lung parenchyma length punctured. This finding is in accordance with previously published data^21,24^.

Our analysis identified pulmonary lesion diameter as another predictor of instant and overall pneumothorax after lung biopsy with smaller lesions increasing the risk, another result consistent with earlier findings^17^.

We also found a significantly lower pneumothorax rate when the coaxial puncture technique was used, providing further support for preferring this technique over the direct puncture technique^18^.

Interestingly, our results do not show an impact of the interventionalist’s experience on the likelihood of lung biopsy resulting in pneumothorax, which is contrary to the results reported by Yeow et al.^17^ and Winokur et al.^20^. Our result might be explained by the fact that the risk factors that can be influenced by the interventionist boil down to correct puncture pathway planning as specified in the *CT fluoroscopy and interventional workflow* subsection of Materials and Methods and that it is our policy that the interventionalist generally discusses the planned pathway with an experienced colleague before the intervention. All biopsies investigated in this study were performed by radiologists who started their interventional career during the observed time period, generally radiologists in their 5th year of residency to obtain board certification. This setting allowed us to estimate the interventionalist’s experience based on the number of lung biopsies performed before the one included in the analysis.

In a separate analysis of patients with %LAA-950 greater than 5%, we found an instant pneumothorax rate of 49% versus 23% in those with scores of 5% or less. This nicely illustrates the key message that pulmonary emphysema is a risk factor for the occurrence of pneumothorax, but also clearly underlines that we will encounter pneumothorax also in patients with little to no emphysema. Given that pneumothorax is a treatable condition, we therefore think that lung biopsies should not be withheld from patients with pulmonary emphysema as long as there is a clear clinical indication.

Our study has several limitations that need to be discussed.

Automatic segmentation of the lung and emphysema scoring with the density mask technique as used in this retrospective analysis is currently the only method to objectively quantify the extent of emphysematous lung destruction. While the use of this objective and reproducible method is an asset of our study, it has to be pointed out that the emphysema score does not take into account other aspects of emphysema-associated lung destruction such as peribronchial thickening or nonemphysematous lung tissue impairment, which may also affect the likelihood of pneumothorax development during lung biopsy. Therefore, visual scoring, while being more time-consuming and subjective, might ultimately be a better predictor as found in a study predicting mortality in patients with chronic obstructive pulmonary disease (COPD)^25^.

We did not use the same technique for pulmonary tissue sampling in all patients included in our analysis. In the majority of cases, we used the recommended combination of a coaxial needle with a cutting needle and obtained two tissue samples^18,26–28^; however, in some cases we used a larger cutting needle alone and obtained only one tissue sample^29^. Moreover, the needle size varied slightly. The technique and needle size were retrieved from the records and included in the logistic regression and lasso regression analysis. Overall, however, we must be aware that documentation may be incomplete, e.g., with respect to the number of samples taken, and some variations are therefore possibly not accurately captured in this retrospective analysis.

Puncture-site-down positioning of the patient immediately after the intervention was reported to reduce the incidence of biopsy-related pneumothorax requiring chest tube insertion while the incidence of less severe pneumothorax was found to be unaffected^30,31^. Puncture-site-down positioning after lung biopsy is promoted in our department. However, we cannot verify how rigorously it was adhered to by interventionalists. As all interventionalists included in this study started their interventional career during the observed time period and acquired their skills from the physician previously working in the position it is likely, though, that they adopted established procedures and measures.

Furthermore, some authors promote a lateral position of the patient with the lung to be punctured pointing downwards during the biopsy procedure^32,33^. This is a method not generally endorsed at our institution because we think that the position that allows the best access to the pulmonary target lesion and at the same time is most comfortable for the patient, thus ensuring the best compliance and minimising movement, outweigh the potential benefits from a lateral position with the punctured lung pointing downwards. In some cases, the position ensuring the best access to the lesion and the lateral position with the punctured lung pointing downwards might have coincided, resulting in a slightly lower likelihood of pneumothorax. However, these would have been only very few cases and the impact, if at all present, would not have been great.

Lastly, there is some leeway when deciding on the need to insert a chest tube, a decision which is primarily based on the severity of pneumothorax and the patient’s symptoms. In some instances, therefore, another physician might have decided differently. This might render the outcome parameter of chest tube insertion a bit more arbitrary than pneumothorax development as such.

In conclusion, quantitatively determined pulmonary emphysema extent is a positive predictor of the likelihood of pneumothorax development in CT-guided lung biopsy and the need for chest tube insertion.

## Supplementary information

Supplementary file1 (DOCX 13 kb)

Supplementary file2 (DOCX 13 kb)
