# Supplementary material for: Impact of quantitative pulmonary emphysema score on the rate of pneumothorax and chest tube insertion in CT-guided lung biopsies
Source: Sci Rep. 2020 Jul 3;10:10978. doi: 10.1038/s41598-020-67348-0 (PMC7335035; doi:10.1038/s41598-020-67348-0)
Supplement: Supplementary file 1 — Supplementary file1 (DOCX 13 kb) [file 41598_2020_67348_MOESM1_ESM.docx]

|  | Predictive variable | Odds ratio | z | P-value | 95% Conf. Interval |
| --- | --- | --- | --- | --- | --- |
| Instant pneumothorax | Emphysema score (%LAA-950) | 1.0757 | 3.51 | <0.001 | 1.0328-1.1204 |
| Overall pneumothorax | Emphysema score  (%LAA-950) | 1.0541 | 2.98 | 0.003 | 1.0183-1.0913 |
| Chest tube insertion | Emphysema score  (%LAA-950) | 1.0847 | 3.69 | <0.001 | 1.0389-1.1326 |

Table I: Least absolute shrinkage and selection operator (Lasso) regression analysis with control variables to be selected by lassos and 10-fold cross-validation.

%LAA-950: Low-attenuation areas less than -950 Hounsfield units
