# Supplementary material for: Impact of quantitative pulmonary emphysema score on the rate of pneumothorax and chest tube insertion in CT-guided lung biopsies
Source: Sci Rep. 2020 Jul 3;10:10978. doi: 10.1038/s41598-020-67348-0 (PMC7335035; doi:10.1038/s41598-020-67348-0)
Supplement: Supplementary file 2 — Supplementary file2 (DOCX 13 kb) [file 41598_2020_67348_MOESM2_ESM.docx]

|  |  | Emphysema score (%LAA-950) ≤ 5% | Emphysema score (%LAA-950) > 5% |
| --- | --- | --- | --- |
| Instant pneumothorax | Yes | 73 (23 %) | 29 (49 %) |
|  | No | 239 (77 %) | 30 (51 %) |
|  | Total | 312 (100 %) | 59 (100%) |
| Overall pneumothorax | Yes | 105 (34 %) | 35 (59%) |
|  | No | 207 (66 %) | 24 (41%) |
|  | Total | 312 (100 %) | 59 (100%) |
| Chest tube inseertion | Yes | 23 (7%) | 12 (20%) |
|  | No | 288 (93%) | 47 (80%) |
|  | Total | 311 (100%) | 59 (100%) |

Table II: Descriptive statistics of subgroup analysis of patients with %LAA-950 ≤ 5% and those with %LAA-950 > 5%.

%LAA-950: Low-attenuation areas less than -950 Hounsfield units
